# The impact of a healthy lifestyle on the risk of esophageal and gastric cancer subtypes

**DOI:** 10.1007/s10654-022-00899-w

**Published:** 2022-08-19

**Authors:** Piet A. van den Brandt

**Affiliations:** 1 Department of Epidemiology, GROW - School for Oncology and Reproduction, Maastricht University Medical Centre, PO Box 616, 6200 MD Maastricht, The Netherlands; 2 Department of Epidemiology, CAPHRI - School for Public Health and Primary Care, Maastricht University Medical Centre, PO Box 616, 6200 MD Maastricht, The Netherlands

**Keywords:** Esophageal cancer, Gastric cancer, Healthy lifestyle, Cohort study

## Abstract

Few prospective studies have been conducted on a combined healthy lifestyle and risk of esophageal and gastric cancer, and even less on subtypes: esophageal squamous cell carcinoma (ESCC), esophageal adenocarcinoma (EAC), gastric cardia adenocarcinoma (GCA), and gastric non-cardia adenocarcinoma (GNCA). The relationship of a healthy lifestyle score (HLS) with risk of these cancers was investigated in the Netherlands Cohort Study. In 1986, 120,852 men and women aged 55–69 years provided information on dietary and lifestyle habits. The HLS was derived from information on smoking, body mass index, physical activity, Mediterranean diet adherence, and alcohol intake. After 20.3 years of follow-up, multivariable case-cohort analyses were based on 333 incident esophageal and 777 gastric cancer cases, and 3720 subcohort members with complete data on lifestyles and confounders. The impact of changing to healthy lifestyles was estimated with the rate advancement period (RAP). The HLS was significantly inversely associated with risk of esophageal and gastric cancer, and subtypes (except EAC), in a linear fashion. The observed HR decrease per 1-point increase in HLS was 31% for esophageal, and 19% for gastric cancer, 49% for ESCC, 23% for GCA, and 18% for GNCA. The RAP per 1-point increase in HLS ranged from − 11.75 years for ESCC to − 2.85 years for GNCA. Also after excluding smoking, inverse associations between the HLS and esophageal and gastric cancer risk were still apparent. These results suggest that adhering to a combination of healthy modifiable lifestyle factors may substantially reduce the risk of esophageal and gastric cancer.

**Supplementary Information:**

The online version contains supplementary material available at 10.1007/s10654-022-00899-w.

## Introduction

Esophageal and gastric cancer belong to the most frequently diagnosed cancers worldwide, while the prognosis of both cancer types is still very poor. Regarding incidence, gastric cancer was the fifth most common cancer worldwide in 2020, while esophageal cancer ranked seventh; for mortality these ranks were four and six, respectively [1]. Based on histologic subtyping, esophageal cancer can be subdivided into esophageal squamous cell carcinoma (ESCC) and esophageal adenocarcinoma (EAC); gastric cancer is topographically subtyped into gastric cardia adenocarcinoma (GCA), and gastric non-cardia adenocarcinoma (GNCA). The incidence rates of EAC and GCA have increased considerably in the United States [2, 3] and Europe since the 1980s [4, 5]. Esophageal cancer is specifically common in Eastern and South Central Asia, and Southern and Eastern Africa. Typically, in these high risk populations the majority is of the ESCC type, in contrast to low risk populations, where EAC is more common [1]. Gastric cancer incidence rates are highest in Eastern Asia, Latin America and Eastern Europe (often GNCA type), and low in North-America and Northern Europe [1].

Primary prevention of cancer by lifestyle and dietary modifications offers an important strategy to reduce the population burden of many cancers and thus remains high priority. Lifestyle factors such as tobacco smoking, alcohol consumption, diet, physical activity, and relative weight (Body Mass Index, BMI) are considered important modifiable factors for preventing cancer. While the individual roles of these lifestyle factors in cancer risk have been extensively documented [6–8], little is known about their joint effects. Prospective studies that investigated combinations of the abovementioned modifiable lifestyle factors have reported a reduced risk of total cancer [9] when comparing subjects with more healthy lifestyles to those with less healthy lifestyles. The impact of a combined healthy lifestyle on risk of specific cancers has also been studied, but mostly for colorectal and breast cancer [9]. Few prospective studies have been conducted on a combined healthy lifestyle and risk of esophageal and gastric cancer, and even less on subtypes of these cancers, while the etiology of these subtypes might differ [10, 11]. Risk factors for esophageal and gastric cancer subtypes are different; for example, while smoking is strongly associated with ESCC and moderately with EAC, GCA and GNCA, alcohol drinking is associated with ESCC, and obesity with EAC and GCA risk [8, 11, 12]. The studies that reported on healthy lifestyle and esophageal or gastric cancer were mostly done in Chinese population cohorts [13–16]; other studied cohorts were the US-based NIH-AARP cohort [17], the European EPIC cohort [18, 19], and the Iranian Golestan cohort [20]. Two studies investigated subtypes of gastric cancer [13, 19]; one study focused on ESCC [20], but no studies were published on EAC.

In these studies, the dietary component of HLS was usually based on very few food items, but Buckland et al. [19] used adherence to the Mediterranean diet to characterize a healthy diet and combined this with other lifestyles to study gastric cancer (subtypes) to find rather strong inverse associations with GCA and GNCA in the EPIC cohort. In other cohorts, MD adherence was also inversely related to risk of gastric cancer subtypes [21] and ESCC [21, 22].

The impact of a healthy lifestyle score, combining information on smoking, BMI, physical activity, alcohol intake, and Mediterranean diet adherence, on the risk of esophageal and gastric cancer (subtypes) was investigated in the Netherlands Cohort Study (NLCS). We previously found inverse associations between Mediterranean diet adherence (excluding alcohol) and risk of these cancers in the NLCS [21]. Analyses were performed for overall esophageal and gastric cancer, to enable comparison with the available literature, and for subtypes of these cancers.

## Methods

### Study design and cancer follow-up

The NLCS started in September 1986 and included 58,279 men and 62,573 women aged 55–69 years [23]. At baseline, participants completed a mailed, self-administered questionnaire on cancer risk factors. The NLCS study was approved by institutional review boards from Maastricht University and the Netherlands Organization for Applied Scientific Research. All cohort members consented to participation by completing the questionnaire. For data processing and analysis the case-cohort method was used [24]. Accumulated person-years in the cohort were estimated from a subcohort (n = 5000; 2411 men and 2589 women), randomly sampled from the cohort immediately after baseline. These subcohort members were actively followed up biennially for vital status information using population registries.

Follow-up for cancer incidence was established by annual record linkage with the Netherlands Cancer Registry and PALGA, the nationwide Dutch Pathology Registry [25]. Completeness of follow-up through record linkage with cancer registries and PALGA was estimated to be greater than 95% [26]. During 20.3 years of follow-up (September 17, 1986 until December 31, 2006), 164 ESCC, 259 EAC, 254 GCA, and 741 GNCA cases without prevalent cancer (except skin cancer) at baseline were detected. Histology codes for esophageal cancer (ICD-O-3 code C15) included 8050–8076 for ESCC, and 8140–8141, 8190–8231, 8260–8263, 8310, 8430, 8480–8481, 8490, 8560, and 8570–8572 for EAC. For gastric cancer (ICD-O-3 code C16), histology codes were C16.0 for GCA and C16.1–16.9 for GNCA. After excluding participants with prevalent cancer (except skin cancer) at baseline from the subcohort, 4774 subcohort members remained. Participants with incomplete or inconsistent dietary data, or missing values for the other considered lifestyle factors and predefined confounders were excluded from the analysis. In the current analysis, 133 ESCC, 200 EAC, 191 GCA, and 586 GNCA cases, and 3,720 subcohort members were included (Supplementary Figure S1).

### Exposure assessment

The 11-page baseline questionnaire measured dietary intake (including alcohol), detailed smoking habits, anthropometry, physical activity and other risk factors related to cancer [23]. Habitual consumption of food and beverages during the year preceding baseline was assessed using a 150-item semi-quantitative food-frequency questionnaire. The food-frequency questionnaire has been validated and tested for reproducibility [27, 28]. Nutrient intakes were calculated using the computerized Dutch food composition table [29]. Consumption of alcoholic beverages was addressed by questions on beer, red wine, white wine, sherry and other fortified wines, liqueur types containing on average 16% ethanol, and (Dutch) gin, brandy, and whiskey. Respondents who consumed alcoholic beverages less than once a month were considered non-users. Tobacco smoking was addressed through questions on smoking status (never, ex, or current smoker) and inhalation for cigarette, cigar, and pipe smokers. Additional questions were asked on the ages at first and last exposure to smoking, smoking frequency, and duration for cigarette, cigar, and pipe smokers. Information on height (in cm) and weight at baseline (in kg) was also collected using the self-administered questionnaire, from which BMI (weight/height^2^) was calculated in kg/m^2^. Non-occupational physical activity was calculated by adding the minutes spent per day on cycling or walking, shopping, walking the dog, gardening, and sports or exercise as reported previously [30].

### Mediterranean diet score

Adherence to the MD was assessed using the alternate Mediterranean Diet Score (aMED) [31, 32], which is an adapted version of the traditional Mediterranean Diet Score created by Trichopoulou et al. [33, 34]. The aMED contains 9 dietary components that are typical of the Mediterranean diet. To control for energy intake, the intake of each component was first adjusted to a daily intake of 2000 kcal [31, 32, 34]. For each of the presumed beneficial food items [vegetables (without potatoes), legumes, fruits, nuts, whole grains, fish, and the ratio of monounsaturated to saturated fatty acid intake (MUFA:SFA)], one point was given when the intake was at least the sex-specific median intake, and zero otherwise. For red and processed meat, 1 point was given (and 0 otherwise) when the intake was below the sex-specific median intake. In the full aMED, 1 additional point is normally given when alcohol intake is between 5 and 25 g/day, and 0 otherwise [32]. However, since alcohol is a risk factor for esophageal and probably also gastric cancer [35, 36], alcohol was excluded from the score in the present analysis. The reduced 9-point sum score (aMEDr) ranged from zero to eight points (minimal to maximal conformity).

### Healthy lifestyle score

Following a previous analysis of a combined healthy lifestyle score and mortality [37] (in which binary scores were used for adherence to component lifestyles), a more refined healthy lifestyle score was constructed to appraise a higher proportion of the variability in the population, as done by Romaguera et al. [18]. In this healthy lifestyle score (HLS), scores for five modifiable lifestyle factors (BMI, smoking, physical activity, Mediterranean diet adherence, and alcohol intake) were combined, while each component factor was scored on three levels, representing full (score 1), partial (0.5) and noncompliance (0) with the public health recommendation for that component. Table S1 shows the cutoffs and scores for each of these five factors. The cutoffs for physical activity and alcohol were in line with recommendations from the Health Council of the Netherlands [38, 39]. The combined sum score ranged from zero to five points (minimal to maximal healthy lifestyle).

### Statistical analysis

Hazard ratios (HRs) and 95% confidence intervals (95% CIs) for associations of the combined healthy lifestyle score with incidence of esophageal and gastric cancer subtypes were estimated using Cox proportional hazards models with follow-up duration as time variable. Person-years at risk for subcohort members were calculated from baseline until diagnosis of esophageal or gastric cancer, death, emigration, loss to follow-up or end of follow-up, whichever came first. Standard errors were estimated using the Huber-White sandwich estimator to account for the increased variance because of subcohort sampling [40]. It was verified that the proportional hazards assumption was not violated using scaled Schoenfeld residuals [41] and − ln(− ln) survival plots. Because most previous studies published on overall esophageal or gastric cancer, results for these outcomes are presented (enabling literature comparisons) next to subtypes of esophageal and gastric cancer.

The associations between the HLS and risk of esophageal and gastric cancer subtypes were investigated on a categorical and continuous scale in survival analyses. We combined both sexes, because of the limited number of cases and because no statistically significant interaction by sex was found. The HLS score was categorized based on the distribution in the subcohort into 6 categories: 0–1.5, 2, 2.5, 3, 3.5, and 4–5 points. Participants with a HLS of 2 points formed the reference group in categorical analyses, because category 0–1.5 was too small. Tests for trends were assessed by assigning median values of the lifestyle score in the subcohort to the exposure categories and fitting these as continuous terms in the regression models. In the continuous analyses, HRs were estimated per increment of 1 point.

In multivariable-adjusted survival analyses, the associations were adjusted for the following predefined (literature-based) confounders, which were included in the final multivariable-adjusted model independent of their effect on the estimated HRs: age at baseline (years; continuous), sex (male/female), smoking frequency (number of cigarettes per day; continuous, centered), and duration (number of years; continuous, centered), highest level of education [primary school or lower vocational (low), secondary school or medium vocational (medium), and higher vocational or university (high)], total energy intake (kcal/day; continuous), family history of esophageal (for esophageal cancer subtypes) or gastric cancer (for gastric cancer subtypes). Because a recent Canadian cohort study showed only inverse associations with HLS for participants without chronic conditions at baseline [42], we also included history of chronic diseases at baseline [physician-diagnosed myocardial infarction, angina pectoris, stroke, hypertension, diabetes, asthma or bronchitis (no, yes)] as covariate.

For the analyses regarding the healthy lifestyle score, the association between each of the component lifestyle factors and cancer risk was evaluated first in Cox regression, while controlling for age, education, energy intake, family history of esophageal/ gastric cancer, history of chronic diseases, and additionally for the other lifestyle components.

The distribution of the subcohort members by the combined healthy lifestyle score and various characteristics was examined by cross-tabulations and summary statistics, for men and women. Distributions of general characteristics for esophageal and gastric cancer subtype cases, as well as subcohort members, were calculated as frequencies for categorical variables and means with standard deviations for continuous variables.

To further investigate the dose–response relations between the healthy lifestyle score and risk of esophageal and gastric cancer subtypes, restricted cubic splines with three knots were used to graphically present the dose–response curves without making a priori assumptions about their shapes. Wald tests were performed to evaluate the linearity of these relationships.

In addition to the main analyses of the healthy lifestyle score and risk of cancer subtypes, analyses were also stratified by age, sex, level of education, family history of esophageal (or gastric) cancer, and history of chronic diseases. Interactions with these factors were tested using Wald tests and cross-product terms. Because of low cancer case numbers, these analyses were conducted using continuous lifestyle scores. In sensitivity analyses, analyses were repeated after excluding cancers (and person-years) occurring in the first two years of follow-up, and also after splitting the follow-up period in three periods.

Because of the importance of smoking as a cancer risk factor, an additional analysis was done with a combined healthy lifestyle score, which excluded smoking, to evaluate the importance of lifestyle factors other than smoking.

The impact of a change in lifestyle score was estimated using the rate advancement period (RAP) [43], by dividing the regression coefficient of the lifestyle score by the regression coefficient of age. The linearity between the log(hazard) and age was verified using restricted cubic splines regression. Confidence intervals for RAP were calculated using variance and covariance estimates for the regression coefficients [43].

Population attributable fractions were calculated [44] to estimate the potentially avoidable proportion of cancer if all participants would shift towards the healthiest lifestyle category. The STATA-command “punafcc” was used to calculate the population attributable fractions and 95% CIs [45]. Analyses were performed using Stata version 14; presented *P *values are two-sided, with *P* < 0.05 considered as statistically significant.

## Results

The mean (SD) score of the combined healthy lifestyle score (HLS) among subcohort members was 3.0 (0.9); for men it was 2.8 (0.9) and for women 3.3 (0.8). Table 1 summarizes several baseline characteristics by healthy lifestyle score in male and female subcohort members. Age and energy intake were not related to the healthy lifestyle score in men and women. While the associations between the healthy lifestyle score and alcohol intake, aMEDr score, BMI, and smoking were as expected considering the score composition, women with a high score were somewhat more often highly educated. Women in the lowest category of the healthy lifestyle score more often reported a history of chronic diseases. Family history of esophageal or gastric cancer seemed not clearly related to the score.

**Table 1:** Baseline characteristics (means, or percent) by combined healthy lifestyle score (HLS) in male and female subcohort members with complete dietary and covariable data, Netherlands Cohort Study

Characteristic	Combined healthy lifestyle score (points), categories					
	0–1.5	2	2.5	3	3.5	4–5
**Men**						
*Median score (pts)*	*1.5*	*2.0*	*2.5*	*3.0*	*3.5*	*4.0*
N	199	293	378	388	336	240
Age, mean (yr)	61.4	60.9	61.1	61.4	61.1	61.4
Energy intake (kcal/day)	2186	2233	2167	2148	2164	2103
Alcohol intake (g/day)	31.8	23.6	16.2	11.3	8.0	4.7
aMEDr^a^ score (points)	2.8	3.2	3.6	3.9	4.5	5.4
Physical activity, nonoccupational (min/day)	36.0	60.9	73.6	91.8	99.7	110.9
BMI (kg/m^2^)	26.4	25.5	25.2	24.7	24.3	23.9
Height (cm)	176.6	176.2	177.0	176.3	176.8	176.6
Never smoker (%)	0.0	1.4	2.9	8.0	13.4	38.8
Ever cigarette smokers only						
Smoking frequency (cigarettes/day)	21.1	18.5	17.8	16.7	15.2	13.8
Smoking duration (years)	38.1	36.5	34.3	33.1	30.6	26.4
University or higher vocational education (%)	20.6	22.2	19.0	19.1	20.5	21.7
Family history of esophageal cancer (%)	0.5	1.4	0.5	0.5	0.6	1.3
Family history of gastric cancer (%)	8.0	4.8	5.8	7.0	8.9	7.5
History of selected chronic diseases^b^ (%)	44.7	41.0	39.4	46.4	43.2	41.7
**Women**						
*Median score (pts)*	*1.5*	*2.0*	*2.5*	*3.0*	*3.5*	*4.0*
N	73	131	270	394	425	593
Age, mean (yr)	61.6	60.8	61.5	61.6	61.4	61.3
Energy intake (kcal/day)	1682	1677	1648	1689	1684	1697
Alcohol intake (g/day)	18.2	10.9	8.4	6.8	4.3	2.9
aMEDr^a^ score (points)	2.8	3.0	3.3	3.6	4.1	5.0
Physical activity, nonoccupational (min/day)	30.0	37.1	43.4	60.5	67.5	88.1
BMI (kg/m^2^)	26.7	26.3	26.0	25.5	25.0	23.8
Height (cm)	164.0	164.9	164.9	165.1	165.4	165.9
Never smoker (%)	1.4	16.0	35.2	52.8	67.1	84.5
Ever cigarette smokers only						
Smoking frequency (cigarettes/day)	16.8	13.5	11.5	10.9	9.8	9.5
Smoking duration (years)	35.3	32.0	29.6	26.9	23.8	21.6
University or higher vocational education (%)	9.6	7.6	7.4	11.2	8.0	11.0
Family history of esophageal cancer (%)	1.4	0.0	1.1	0.0	1.4	1.2
Family history of gastric cancer (%)	8.2	6.9	4.8	8.1	4.7	6.9
History of selected chronic diseases^b^ (%)	43.8	39.7	41.5	39.8	36.7	36.3

^a^aMEDr: alternate Mediterranean diet score excluding alcohol

^b^Chronic diseases at baseline: myocardial infarction, angina pectoris, stroke, hypertension, diabetes, asthma or bronchitis (no, yes)

Table 2 shows baseline characteristics of cancer cases and subcohort members, for men and women together, because there was no significant interaction by sex in later Cox regression analyses. Cases with esophageal or gastric cancer (subtypes) had lower HLS scores than subcohort members; this was observed for both men and women (data not shown). On average, cases were older, more often were males, smokers with higher smoking frequency and duration, had a family history of esophageal or gastric cancer, were longer, had a higher alcohol intake and lower aMEDr score than subcohort members (Table 2).

**Table 2:** Baseline characteristics (means, or percent) of subcohort members and cancer cases, Netherlands Cohort Study

Characteristic	Subcohort	Esophageal cancer cases			Gastric cancer cases		
		Overall	ESCC	EAC	Overall	GCA	GNCA
N	3720	333	133	200	777	191	586
Women (%)	50.7	30.0	42.9	21.5	29.5	17.3	33.4
Age at baseline (yr), mean	61.3	61.7	62.1	61.4	62.0	61.3	62.2
Energy intake (kcal/day)	1922	2006	1918	2064	2033	2074	2020
Alcohol intake (g/day)	10.5	17.4	22.0	14.3	12.8	14.2	12.3
aMEDr^a^ score (points)	4.0	3.8	3.5	4.0	3.7	3.7	3.6
Physical activity, nonoccupational (min/day)	73.1	73.6	68.2	77.3	80.3	84.0	79.1
BMI (kg/m^2^)	25.0	25.3	24.3	25.9	25.1	25.7	24.9
Never smoker (%)	34.8	17.7	16.5	18.5	19.7	14.7	21.3
Ever cigarette smokers only							
Smoking frequency (cigarettes/day)	15.4	19.0	18.2	19.6	16.9	18.1	16.4
Smoking duration (years)	31.6	34.6	36.8	33.1	34.2	33.7	34.3
University or higher vocational education (%)	14.9	15.6	13.5	17.0	12.9	20.4	10.4
Family history of esophageal cancer (%)	0.8	2.1	1.5	2.5	1.2	1.0	1.2
Family history of gastric cancer (%)	6.7	7.2	7.5	7.0	10.8	8.4	11.6
History of selected chronic diseases^b^ (%)	40.5	42.9	40.6	44.5	39.8	42.4	38.9
Combined healthy lifestyle score (HLS, points), mean	3.0	2.7	2.5	2.8	2.8	2.7	2.8
Combined HLS (points), categories							
0–1.5 pts	7.3	15.6	21.8	11.5	10.3	13.6	9.2
2	11.4	18.0	19.5	17.0	17.1	14.7	17.9
2.5	17.4	19.5	22.6	17.5	21.2	23.0	20.6
3	21.0	18.9	16.5	20.5	20.3	22.0	19.8
3.5	20.5	15.6	9.8	19.5	17.6	16.2	18.1
4–5	22.4	12.3	9.8	14.0	13.4	10.5	14.3

*ESCC* esophageal squamous cell carcinoma, *EAC* esophageal adenocarcinoma, *GCA* gastric cardia adenocarcinoma, *GNCA* gastric non-cardia adenocarcinoma, *HLS* healthy lifestyle score

^a^aMEDr: alternate Mediterranean diet score excluding alcohol

^b^Chronic diseases at baseline: myocardial infarction, angina pectoris, stroke, hypertension, diabetes, asthma or bronchitis (no, yes)

**Fig. 1:**
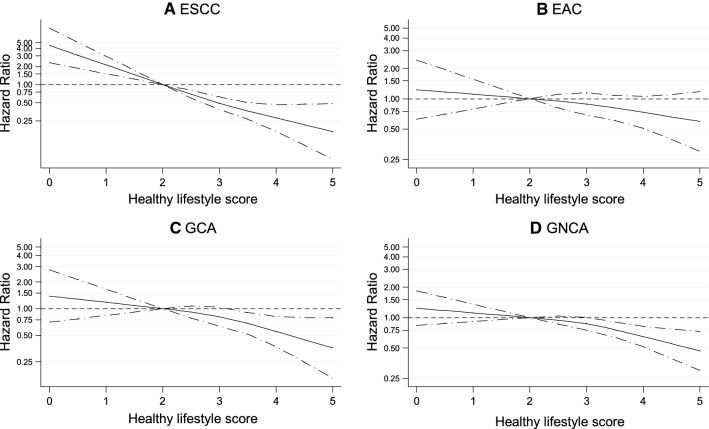
Spline regression curves for the association between healthy lifestyle score (HLS) and risk of **A** esophageal squamous cell carcinoma, **B** esophageal adenocarcinoma, **C** gastric cardia adenocarcinoma, and **D** gastric non-cardia adenocarcinoma, Netherlands Cohort Study (NLCS). Solid lines represents point estimates and dashed lines represent 95% confidence intervals. Multivariable HRs were calculated by restricted cubic spline regression (using 3 knots) adjusting for: age at baseline (years; continuous), sex, cigarette smoking frequency (number of cigarettes per day; continuous, centered) and duration (number of years; continuous, centered), highest level of education (primary school or lower vocational, secondary or medium vocational, and higher vocational or university), family history of esophageal cancer, family history of gastric cancer (respectively), chronic diseases at baseline: myocardial infarction, angina pectoris, stroke, hypertension, diabetes, asthma or bronchitis (no, yes), energy intake (continuous, kcal/day). *P*-values for non-linearity tests were 0.596 for ESCC, 0.744 for EAC, 0.466 for GCA and 0.268 for GNCA. Abbreviations: ESCC, esophageal squamous cell carcinoma; EAC, esophageal adenocarcinoma; GCA, gastric cardia adenocarcinoma; GNCA, gastric non-cardia adenocarcinoma; HLS, healthy lifestyle score

Table 3 shows results of the multivariable-adjusted analyses of the associations of the healthy lifestyle score with risk of overall esophageal and gastric cancer, and their subtypes. The HLS was statistically significantly inversely associated with risk of overall esophageal (*P*-trend < 0.001) and gastric cancer (*P*-trend < 0.001). Supplementary Figures S2 and S3 show sex-specific results of categorical analyses for esophageal and gastric cancer, respectively, indicating that the HLS was significantly inversely associated with both cancer types in men and women. The HLS was also significantly inversely associated with risk of ESCC, GCA and GNCA (all with *P*-trend < 0.008, Table 3). Only EAC did not show a statistically significant inverse association with HLS. For all cancer types, the interaction with sex was not statistically significant, with all *P*-values for interaction > 0.127 (Table 3). The inverse associations were rather strong: compared to the reference category of 2 points, participants with a HLS of 4–5 points had a Hazard Ratio (HR) (and 95% Confidence interval, CI) of 0.45 (0.29, 0.70) for esophageal and 0.52 (0.38, 0.71) for gastric cancer. The corresponding HR (95% CI) was 0.25 (0.13, 0.52) for ESCC, and 0.50 (0.35, 0.70) for GNCA. For EAC and GCA, this same comparison yielded moderately inverse, but nonsignificant HRs (95% CI) of 0.65 (0.37, 1.13) and 0.60 (0.33, 1.11), respectively. Cubic spline analyses showed no statistically significant tests for nonlinearity between HLS and cancer risk (Fig. 1 and Supplementary Figure S4). When HLS was modelled as continuous variable in a linear fashion, this again showed statistically significant inverse associations, except for EAC. Per HLS increment of 1 point, the HRs ranged from 0.51 for ESCC to 0.86 for EAC (Table 3).

**Table 3:** Hazard Ratio of esophageal and gastric cancer (subtypes), according to healthy lifestyle score in multivariable-adjusted^a^ analyses, Netherlands Cohort Study

	Healthy lifestyle score (HLS, points)						*P*-trend	*P*-interaction by sex	Continuous, per 1 point	RAP (95% CI) per 1 point (years)
	0–1.5 pts	2 (Ref)	2.5	3	3.5	4–5				
*Esophageal cancer*										
Person-years in subcohort	4087	6773	10,884	13,258	13,008	14,896			62,905	
No. of cases	52	60	65	63	52	41			333	
Multivariable-adjusted HR	1.28	1	0.76	0.64	0.60	0.45	< 0.001	0.262	0.69	− 7.46
(95% CI)	(0.84–1.95)		(0.52–1.11)	(0.43–0.95)	(0.39–0.90)	(0.29–0.70)			(0.60–0.80)	(− 12.68, − 2.24)
*Gastric cancer*										
No. of cases	80	133	165	158	137	104			777	
Multivariable-adjusted HR	0.92	1	0.85	0.70	0.68	0.52	< 0.001	0.904	0.81	− 3.58
(95% CI)	(0.66–1.28)		(0.65–1.12)	(0.53–0.93)	(0.51–0.90)	(0.38–0.71)			(0.73–0.89)	(− 5.49, − 1.67)
*ESCC*										
No. of cases	29	26	30	22	13	13			133	
Multivariable-adjusted HR	1.62	1	0.76	0.47	0.30	0.26	< 0.001	0.685	0.51	− 11.75
(95% CI)	(0.92–2.85)		(0.44–1.32)	(0.26–0.86)	(0.15–0.61)	(0.13–0.52)			(0.41–0.63)	(− 21.55, − 1.95)
*EAC*										
No. of cases	23	34	35	41	39	28			200	
Multivariable-adjusted HR	1.00	1	0.75	0.78	0.85	0.65	0.188	0.337	0.86	− 3.38
(95% CI)	(0.57–1.78)		(0.45–1.23)	(0.48–1.29)	(0.51–1.42)	(0.37–1.13)			(0.72–1.03)	(− 8.45, − 1.69)
*GCA*										
No. of cases	26	28	44	42	31	20			191	
Multivariable-adjusted HR	1.39	1	1.14	0.98	0.83	0.60	0.008	0.128	0.77	− 8.75
(95% CI)	(0.79–2.46)		(0.69–1.88)	(0.58–1.65)	(0.48–1.43)	(0.33–1.11)			(0.64–0.92)	(− 21.00, 3.49)
*GNCA*										
No. of cases	54	105	121	116	106	84			586	
Multivariable-adjusted HR	0.79	1	0.77	0.63	0.63	0.50	< 0.001	0.721	0.82	− 2.85
(95% CI)	(0.54–1.15)		(0.57–1.05)	(0.46–0.86)	(0.46–0.87)	(0.35–0.70)			(0.74–0.91)	(− 4.54, − 1.16)

*ESCC* esophageal squamous cell carcinoma, *EAC* esophageal adenocarcinoma, *GCA* gastric cardia adenocarcinoma, *GNCA* gastric non-cardia adenocarcinoma, *HLS* healthy lifestyle score, *HR* hazard ratio, *RAP* rate advancement period

^a^Multivariable analyses adjusted for: age at baseline (years; continuous), sex, cigarette smoking frequency (number of cigarettes per day; continuous, centered) and duration (number of years; continuous, centered), highest level of education (primary school or lower vocational, secondary or medium vocational, and higher vocational or university), family history of esophageal cancer, family history of gastric cancer (respectively), chronic diseases at baseline: myocardial infarction, angina pectoris, stroke, hypertension, diabetes, asthma or bronchitis (no, yes), energy intake (continuous, kcal/day)

The Rate Advancement Period (RAP) per 1-point increase in HLS was estimated at − 7.46 years (95%CI, − 12.68, − 2.24) for overall esophageal cancer, and − 3.58 years (95%CI, − 5.49, − 1.67) for overall gastric cancer (Table 3). The estimated RAPs per 1-point increase in HLS were − 11.75 years for ESCC, − 3.38 for EAC, − 8.75 years for GCA, and − 2.85 years for GNCA.

Estimation of the population attributable fractions (PAFs) suggested that 37.4% (95%CI, 15.1%, 53.9%) of overall esophageal cancer and 30.2% (95%CI, 14.8%, 42.8%) of overall gastric cancer could be avoided if all participants would shift towards the healthiest HLS category. Estimated PAFs were 56.8% (95%CI, 26.9%, 74.5%) for ESCC, 20.5% (95%CI, − 14.9%, 45.0%) for EAC, 37.4% (95%CI, 4.4%, 59.0%) for GCA, and 28.0% (95%CI, 10.4%, 42.2%) for GNCA.

**Fig. 2:**
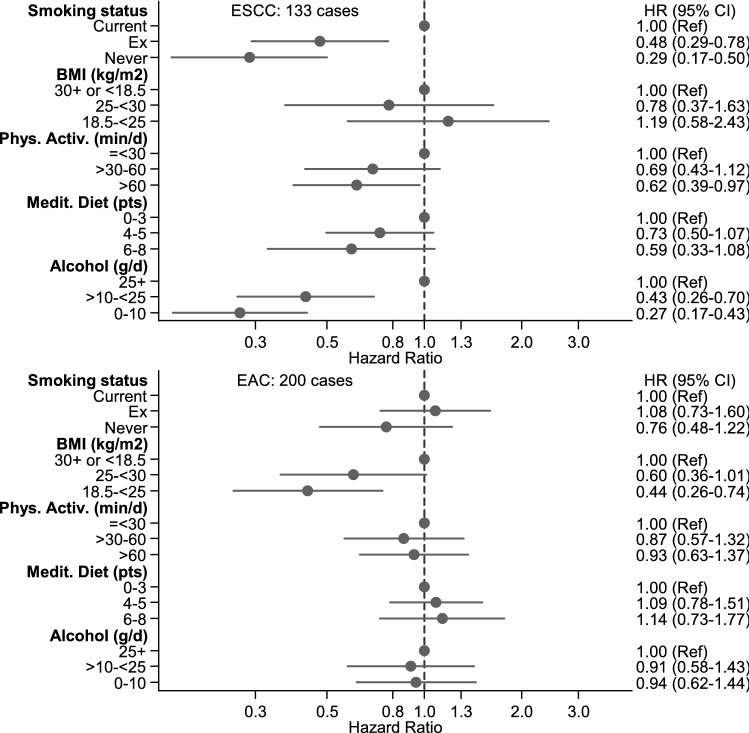
Hazard ratios and 95% CIs (error bars) for the association between risk of ESCC and EAC respectively, with each of the component lifestyle factors of the HLS (with mutual adjustment for the other component lifestyle factors), Netherlands Cohort Study (NLCS). Multivariable HRs were adjusted for: age at baseline (years; continuous), sex, cigarette smoking frequency (number of cigarettes per day; continuous, centered) and duration (number of years; continuous, centered), highest level of education (primary school or lower vocational, secondary or medium vocational, and higher vocational or university), family history of esophageal cancer, chronic diseases at baseline: myocardial infarction, angina pectoris, stroke, hypertension, diabetes, asthma or bronchitis (no, yes), energy intake (continuous, kcal/day), other component lifestyle factors of the HLS. *P*-values for trend tests were for ESCC: < 0.001 for smoking status, 0.145 for BMI, 0.048 for physical activity, 0.042 for Mediterranean Diet adherence, and < 0.001 for alcohol. For EAC, *P*-values for trend tests were: 0.358 for smoking status, 0.002 for BMI, 0.853 for physical activity, 0.525 for Mediterranean Diet adherence, 0.843 for alcohol. *Abbreviations*: ESCC, esophageal squamous cell carcinoma; EAC, esophageal adenocarcinoma; HLS, healthy lifestyle score

**Fig. 3:**
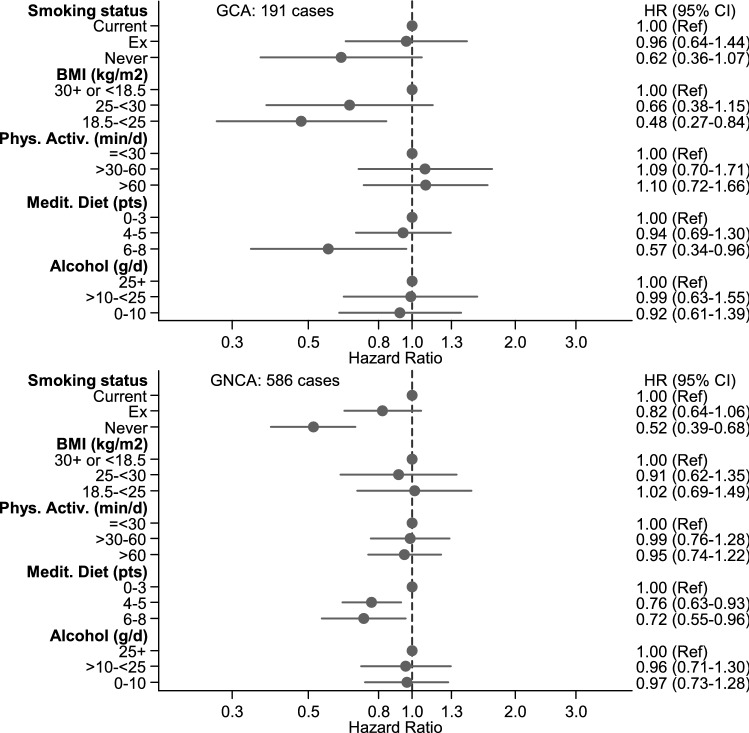
Hazard ratios and 95% CIs (error bars) for the association between risk of GCA and GNCA respectively, with each of the component lifestyle factors of the HLS (with mutual adjustment for the other component lifestyle factors), Netherlands Cohort Study (NLCS). Multivariable HRs were adjusted for: age at baseline (years; continuous), sex, cigarette smoking frequency (number of cigarettes per day; continuous, centered) and duration (number of years; continuous, centered), highest level of education (primary school or lower vocational, secondary or medium vocational, and higher vocational or university), family history of gastric cancer, chronic diseases at baseline: myocardial infarction, angina pectoris, stroke, hypertension, diabetes, asthma or bronchitis (no, yes), energy intake (continuous, kcal/day), other component lifestyle factors of the HLS. *P*-values for trend tests were for GCA: 0.112 for smoking status, 0.006 for BMI, 0.703 for physical activity, 0.055 for Mediterranean Diet adherence, and 0.662 for alcohol. For GNCA, *P*-values for trend tests were: < 0.001 for smoking status, 0.459 for BMI, 0.654 for physical activity, 0.005 for Mediterranean Diet adherence, and 0.845 for alcohol. Abbreviations: GCA, gastric cardia adenocarcinoma; GNCA, gastric non-cardia adenocarcinoma; HLS, healthy lifestyle score

The multivariable-adjusted associations between each of the component lifestyle factors and cancer risk (with mutual adjustment for the other component lifestyle factors) are shown in Fig. 2 for ESCC and EAC, in Fig. 3 for GCA and GNCA, and in Supplementary Figure S5 for overall esophageal and gastric cancer. These analyses show that smoking was significantly associated with risk of overall esophageal and gastric cancer, and ESCC and GNCA; BMI was significantly positively associated with EAC and GCA risk; physical activity was significantly inversely associated with ESCC; MD adherence (aMEDr) was significantly inversely associated with ESCC and all gastric cancer types; alcohol intake was significantly associated with risk of overall esophageal cancer and ESCC.

**Fig. 4:**
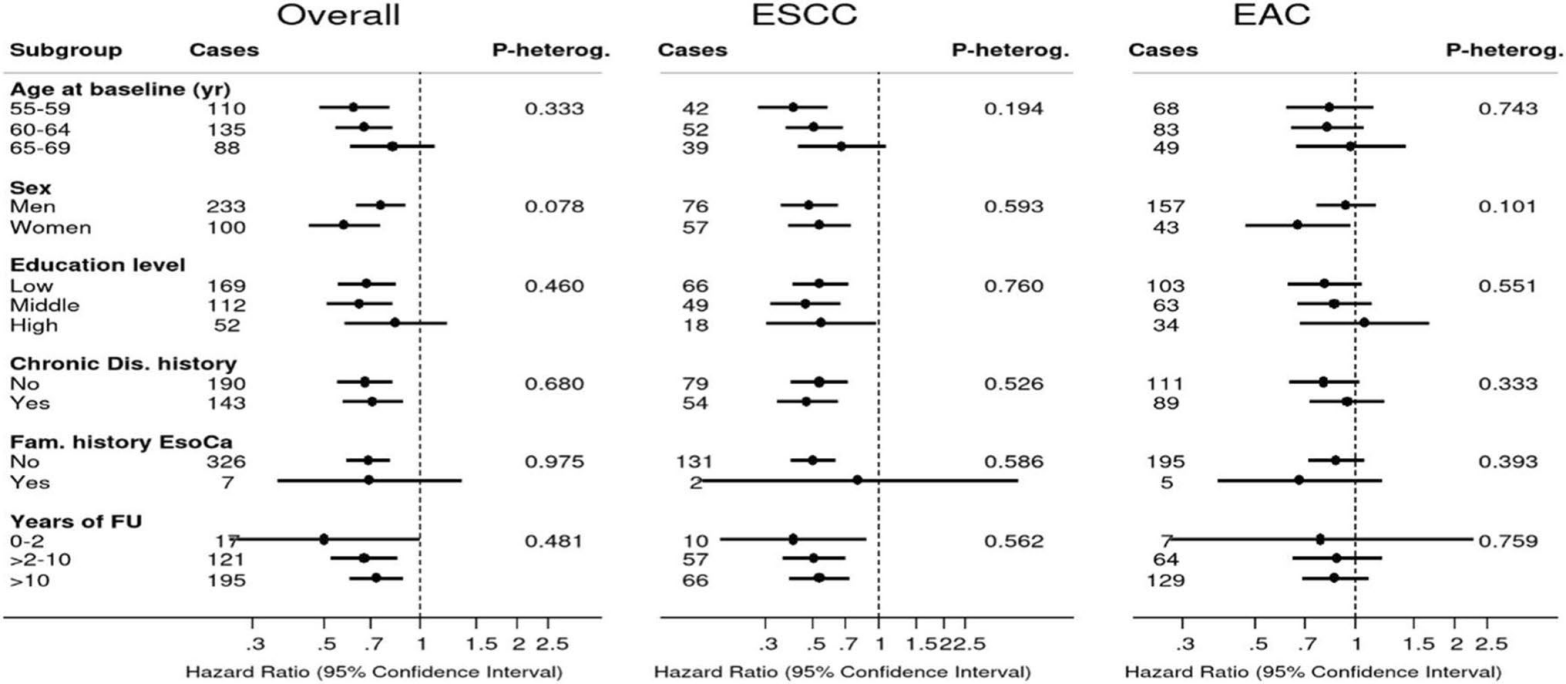
Hazard ratios and 95% CIs (error bars) of esophageal cancer associated with a 1-point increment in healthy lifestyle score (HLS), in subgroups. Multivariable analyses were adjusted for: age at baseline (years; continuous), sex, cigarette smoking frequency (number of cigarettes per day; continuous, centered) and duration (number of years; continuous, centered), highest level of education (primary school or lower vocational, secondary or medium vocational, and higher vocational or university), family history of esophageal cancer, chronic diseases at baseline: myocardial infarction, angina pectoris, stroke, hypertension, diabetes, asthma or bronchitis (no, yes), energy intake (continuous, kcal/day). *Abbreviations*: ESCC, esophageal squamous cell carcinoma; EAC, esophageal adenocarcinoma; HLS, healthy lifestyle score

**Fig. 5:**
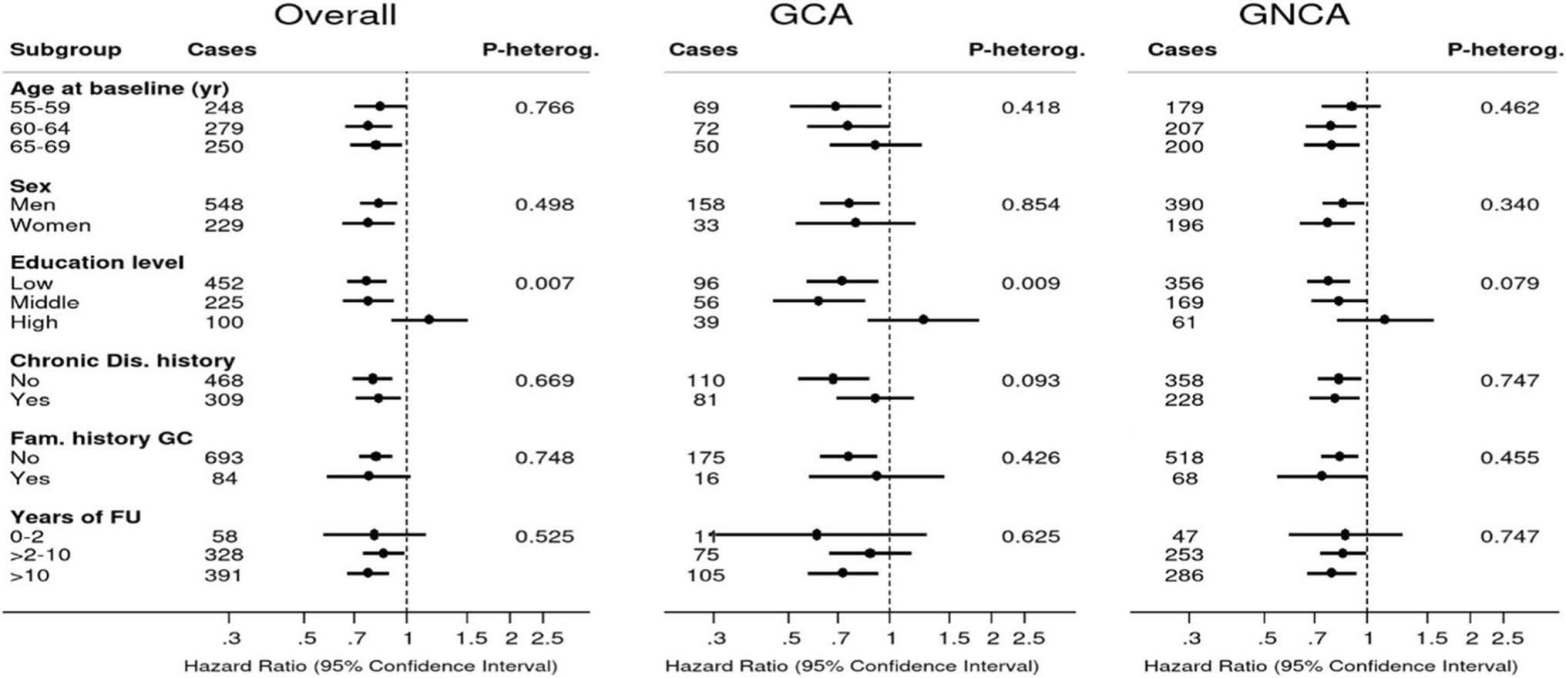
Hazard ratios and 95% CIs (error bars) of gastric cancer associated with a 1-point increment in healthy lifestyle score (HLS), in subgroups. Multivariable analyses were adjusted for: age at baseline (years; continuous), sex, cigarette smoking frequency (number of cigarettes per day; continuous, centered) and duration (number of years; continuous, centered), highest level of education (primary school or lower vocational, secondary or medium vocational, and higher vocational or university), family history of gastric cancer, chronic diseases at baseline: myocardial infarction, angina pectoris, stroke, hypertension, diabetes, asthma or bronchitis (no, yes), energy intake (continuous, kcal/day). Abbreviations: GCA, gastric cardia adenocarcinoma; GNCA, gastric non-cardia adenocarcinoma; HLS, healthy lifestyle score

Figure 4 shows associations between a 1-point increment in HLS and overall esophageal cancer risk, in subgroups of potential effect modifiers: sex, age at baseline, level of education, family history of esophageal cancer, history of chronic diseases. Inverse associations were seen in all subgroups, and there was no significant interaction. Similarly, associations were inverse when the follow-up period was split in 0–2 years, 2–10, and 10–20 years, with no significant interaction (Fig. 4). The corresponding subgroup analyses for ESCC and EAC are also presented in Fig. 4, showing similar patterns of associations with weaker associations in EAC. Figure 5 shows subgroup analyses for gastric cancer (subtypes). Again, no significant interactions were found, except for a significant interaction with level of education (*P*-interaction = 0.007), where an inverse association with HLS was not observed for those with a high level of education. The same pattern was observed for GCA and GNCA subtypes, with a significant interaction for GCA (*P*-interaction = 0.009): the inverse association was most apparent in those with low or middle level of education.

When smoking was omitted from the HLS, the remaining healthy lifestyle score (range 0–4) was significantly associated with risk of overall esophageal cancer and ESCC, and with overall gastric cancer and GCA (Table 4), with no evidence of nonlinearity. There was no significant association with EAC and GNCA. Per 1-point increment of this score excluding smoking, the HRs (95% CI) were 0.75 (0.64, 0.88) and 0.88 (0.79, 0.98) for esophageal and gastric cancer, respectively (Table 4). The corresponding (significant) HRs for ESCC and GCA were 0.61 and 0.79, respectively. Analyses were further stratified by smoking status (two strata, because of low case numbers): never smokers or stopped at least 10 years ago; current smoker or stopped less than 10 years ago (Supplementary Figure S6). These analyses showed inverse associations between the healthy lifestyle score excluding smoking and cancer risk in all subgroups (Supplementary Figure S6), with significant associations for overall esophageal cancer and ESCC in current smokers or those who stopped less than 10 years ago, and for gastric cancer and GNCA among never smokers or those who stopped at least 10 years ago.

**Table 4:** Hazard Ratio of esophageal and gastric cancer (subtypes), according to healthy lifestyle score (excluding smoking) in multivariable-adjusted^a^ analyses, Netherlands Cohort Study

	Healthy lifestyle score without smoking (points)					*P*-trend	*P*-interaction	Continuous,	*P* for
	0–1.5 pts	2 (Ref)	2.5	3	3.5–4		by sex	per 1 point	nonlinearity
*Esophageal cancer*									
Person-years in subcohort	8748	12,587	16,050	15,016	10,504			62,905	
No. of cases	79	83	71	64	36			333	
Multivariable-adjusted HR	1.14	1	0.72	0.76	0.66	0.003	0.242	0.75	0.602
(95% CI)	(0.81–1.61)		(0.51–1.01)	(0.53–1.07)	(0.44–1.00)			(0.64–0.88)	
*Gastric cancer*									
No. of cases	151	180	195	150	101			777	
Multivariable-adjusted HR	1.03	1	0.88	0.77	0.81	0.014	0.770	0.88	0.479
(95% CI)	(0.80–1.34)		(0.70–1.11)	(0.60–0.99)	(0.61–1.06)			(0.79–0.98)	
*ESCC*									
No. of cases	36	38	30	17	12			133	
Multivariable-adjusted HR	1.16	1	0.67	0.45	0.49	< 0.001	0.347	0.61	0.753
(95% CI)	(0.71–1.88)		(0.41–1.11)	(0.25–0.80)	(0.25–0.96)			(0.48–0.77)	
*EAC*									
No. of cases	43	45	41	47	24			200	
Multivariable-adjusted HR	1.13	1	0.75	1.00	0.80	0.289	0.190	0.87	0.890
(95% CI)	(0.72–1.78)		(0.48–1.18)	(0.65–1.55)	(0.48–1.35)			(0.71–1.07)	
*GCA*									
No. of cases	44	43	40	48	16			191	
Multivariable-adjusted HR	1.23	1	0.78	1.07	0.57	0.049	0.502	0.79	0.910
(95% CI)	(0.78–1.94)		(0.50–1.22)	(0.70–1.65)	(0.31–1.02)			(0.65–0.97)	
*GNCA*									
No. of cases	107	137	155	102	85			586	
Multivariable-adjusted HR	0.97	1	0.91	0.68	0.88	0.066	0.958	0.91	0.342
(95% CI)	(0.73–1.30)		(0.70–1.18)	(0.51–0.91)	(0.65–1.19)			(0.81–1.03)	

*ESCC* esophageal squamous cell carcinoma, *EAC* esophageal adenocarcinoma, *GCA* gastric cardia adenocarcinoma, *GNCA* gastric non-cardia adenocarcinoma, *HR* hazard ratio

^a^Multivariable analyses adjusted for: age at baseline (years; continuous), sex, cigarette smoking status, frequency (number of cigarettes per day; continuous, centered) and duration (number of years; continuous, centered), highest level of education (primary school or lower vocational, secondary or medium vocational, and higher vocational or university), family history of esophageal cancer, family history of gastric cancer (respectively), chronic diseases at baseline: myocardial infarction, angina pectoris, stroke, hypertension, diabetes, asthma or bronchitis (no, yes), energy intake (continuous, kcal/day)

## Discussion

In this large prospective study among Dutch men and women aged 55–69 years, a healthy lifestyle score (HLS) which combined nonsmoking, having a normal BMI, being physically active, adhering to a Mediterranean Diet, with no or low alcohol intake, showed a strong statistically significant inverse relationship with risk of esophageal and gastric cancer, in a linear fashion. A one-point increment of the HLS was accompanied by a HR reduction of 31% for overall esophageal, and 19% for gastric cancer. For the cancer subtypes ESCC, GCA and GNCA, associations with the HLS were also significantly inverse, but not significantly for EAC. The observed HR reductions per 1-point increment were 49% for ESCC, 14% for EAC, 23% for GCA, and 18% for GNCA. There was significant interaction with level of education for gastric cancer and GCA; significant inverse associations with HLS were only found in those with a low or medium level of education. These results suggest that adhering to a combination of healthy modifiable lifestyle factors may substantially reduce the risk of esophageal and gastric cancer (subtypes). Also after excluding smoking, inverse associations between the HLS and risk of esophageal and gastric cancer were still apparent.

A recent meta-analysis reported summary HRs (95%CI) for the healthiest versus least healthy combined lifestyle were 0.42 (0.24, 0.75) for esophageal cancer, and 0.60 (0.48, 0.74) for gastric cancer [9]. However, they noted substantial heterogeneity in the published risk estimates, especially for esophageal cancer. In the NLCS, the HR estimates observed for the healthiest lifestyle category in the NLCS for esophageal (HR = 0.45) and gastric cancer (HR = 0.52) are consistent with the meta-analysis estimates.

For esophageal cancer, previous cohort studies reported inverse associations with healthy lifestyle indices combining the components smoking, BMI, physical activity, diet and alcohol [14], or without smoking [17, 18]. The inverse associations with the HLS excluding smoking in the NLCS are in line with those from EPIC [18] and the NIH-AARP [17], with no indication of effect modification by sex. When the full HLS (including smoking) was used, the inverse association became stronger in the NLCS, whereas a Chinese cohort study [14] reported weak associations with a HLS including smoking. Results of studies might differ between regions of the world because one subtype of esophageal and gastric cancers is more predominant in certain countries, and the risk factors might differ between regions.

None of these previous studies looked into esophageal cancer subtypes. In the NLCS, the inverse association was strong and significant in ESCC, while for EAC it was considerably weaker and nonsignificant, with or without smoking as component. The Golestan study [20] is the only other study looking into a subtype (ESCC). They also found a very strong association with a combination of lifestyle and environmental risk factors, but several of these factors seem less relevant for the Western world, e.g. opium smoking, drinking hot tea, tooth loss, indoor air pollution. No other studies have been reported on combined healthy lifestyle and EAC.

Regarding gastric cancer risk, inverse associations with healthy lifestyle indices combining the components smoking, BMI, physical activity, diet and alcohol were found in cohort studies [though not in all [14]] from Singapore and China [13, 15], also for the subtypes GCA and GNCA [13]. Inverse associations were also found when smoking was excluded from the HLS, in the NIH-AARP and EPIC cohorts [17, 18]. Jin et al. [16] observed an inverse association with a HLS combining smoking, alcohol and diet for overall gastric cancer risk in the Chinese Kadoori cohort. Buckland et al. [19] studied overall and subtypes gastric cancer in the EPIC cohort, using adherence to the Mediterranean diet to characterize a healthy dietary pattern. The index combining smoking, alcohol, MD adherence, and BMI (only for GCA) was rather strongly inversely associated with risk of overall gastric cancer, and the subtypes GCA and GNCA, without effect modification by sex. MD adherence was inversely related to risk of esophageal and gastric cancer risk in a recent meta-analysis [46].

As generally observed in previous studies, no significant differences between men and women regarding HLS and esophageal or gastric cancer risk were found in the current study. In the NLCS, a significant interaction was seen between the HLS and level of education for gastric cancer and its subtypes. The inverse association was most apparent in those with low or middle level of education. Few other studies have evaluated interaction with other factors; Buckland et al. [19] found no differences according to educational level or smoking status. In the recent meta-analysis [9] on HLS and cancer risk, subgroup analyses were only conducted for overall cancer. In that analysis, it was also observed that subjects with high educational level show a weaker inverse association between HLS and cancer risk, as compared to those with lower education levels [9]. Also regarding overall cancer, subgroup analyses in a recent Canadian cohort study showed only inverse associations with HLS for participants without chronic conditions at baseline [42]. In the NLCS, no interaction was observed with history of chronic diseases at baseline for esophageal or gastric cancer.

As expected from previous work [8, 12, 21, 35, 36], the component risk factors showed varying associations with esophageal and gastric cancer risk, after mutual adjustment for the remaining components, with notable differences between subtypes. While smoking was positively associated with ESCC and GNCA, BMI was positively associated with EAC and GCA risk. Alcohol was positively, and physical activity inversely associated with ESCC risk. Adherence to MD was inversely related to ESCC risk and all gastric cancer types. These associations are largely in line with the literature [8, 35, 36, 47]. Whereas a significant inverse association between physical activity and ESCC was seen in the NLCS, the few previous studies that reported about physical activity and ESCC found mixed results [48–50].

The combined HLS was significantly associated with all (sub)types except EAC in the NLCS, but the strength of the associations varied, probably reflecting the relative importance of these component risk factors in the etiology of the various subtypes. Also after excluding smoking, the HLS still showed inverse associations in the NLCS, as was found before for overall esophageal and gastric cancer [17, 18].

The Rate Advancement Periods (RAPs) found in the NLCS suggests potentially delayed risks of developing esophageal or gastric cancer (subtypes) when adopting a healthy lifestyle. The RAPs per 1-point increase in HLS varied from − 11.75 years for ESCC to − 2.85 years for GNCA in the NLCS. Only one other cohort study reported RAPs for a HLS combining smoking, alcohol, diet, BMI and physical activity in China [15], and found a lower RAP of − 1.71 per point increase for gastric cancer, compared to − 3.58 years for gastric cancer observed in the NLCS. The RAP estimates the time period by which the risk of cancer could be postponed by adhering to the combined healthy lifestyle. The impact of message to a 60-year-old person with an unhealthy combined lifestyle, that he/she has the same cancer risk as a 70-year-old person with a healthy lifestyle if he/she continues this poor lifestyle (as suggested by a RAP estimate of 10 years) might increase motivation to change lifestyles.

For overall gastric cancer, the estimated PAF (30%) associated with adherence to the healthiest lifestyle in the NLCS was within the range of published PAFs for combined lifestyles, which varied from 19% in EPIC [19] to 48% in Singapore Chinese [13]; for GCA and GNCA, published PAFs [13, 19] were higher than in the NLCS. Whereas a PAF of 37% was found for overall esophageal cancer in the NLCS, no other studies were found with published PAFs associated with combined HLS. For ESCC, the estimated PAF of 57% in the NLCS is lower than the published PAF of 76% in the Golestan cohort [20], but they used a combination of strong risk factors specific for that area. The large variation seen in PAFs associated with combined HLS from different studies can be due to choice of (the number of) considered risk factors in the combination scores, the scoring system and how extreme the chosen categories for least and most healthy lifestyle were defined, and the distribution of the risk factors in the populations studied. This makes it difficult to compare PAFs from different studies.

The prospective design and high completeness of follow-up of the NLCS make information bias and selection bias unlikely. Exclusion of cases diagnosed within the first two years of follow-up also did not change the results. The large cohort with long follow-up enabled cancer subtype-specific analyses. However, case numbers for several subtypes were still low, necessitating combined analyses for men and women to increase the statistical power. The NLCS also has some limitations. Although we adjusted for a large number of potential confounders, residual confounding by unmeasured factors may still exist. For example, no data were available regarding Helicobacter pylori infection, which might have confounded the results in particular for GNCA. The validation study of the food frequency questionnaire has shown that it performs relatively well [27], but measurement error may still have attenuated associations. Because there was no possibility to update dietary intake or other lifestyle data during follow-up, this may have resulted in some attenuated associations too.

In conclusion, this cohort study showed that adhering to a combined healthy lifestyle is rather strongly inversely related to the risk of esophageal and gastric cancer (subtypes). The RAPs per 1-point increase in the healthy lifestyle score varied from − 11.75 years for ESCC to − 2.85 years for GNCA in the NLCS. This suggests that important gains in preventing esophageal or gastric cancer can be made by adhering to a healthy lifestyle.

## Supplementary Information

Below is the link to the electronic supplementary material.

Supplementary file1 (PDF 401 kb)

## References

[1] Sung H, Ferlay J, Siegel RL (2021). Global Cancer Statistics 2020: GLOBOCAN estimates of incidence and mortality worldwide for 36 cancers in 185 countries. CA Cancer J Clin.

[2] Devesa SS, Blot WJ, Fraumeni JF (1998). Changing patterns in the incidence of esophageal and gastric carcinoma in the United States. Cancer.

[3] Trivers KF, Sabatino SA, Stewart SL (2008). Trends in esophageal cancer incidence by histology, United States, 1998–2003. Int J Cancer.

[4] Botterweck AA, Schouten LJ, Volovics A, Dorant E, van Den Brandt PA (2000). Trends in incidence of adenocarcinoma of the oesophagus and gastric cardia in ten European countries. Int J Epidemiol.

[5] Steevens J, Botterweck AA, Dirx MJ, van den Brandt PA, Schouten LJ (2010). Trends in incidence of oesophageal and stomach cancer subtypes in Europe. Eur J Gastroenterol Hepatol.

[6] Secretan B, Straif K, Baan R (2009). A review of human carcinogens–Part E: tobacco, areca nut, alcohol, coal smoke, and salted fish. Lancet Oncol.

[7] IARC. IARC Working Group on the Evaluation of Carcinogenic Risks to Humans. Personal habits and indoor combustions. Volume 100 E. A review of human carcinogens. IARC Monogr Eval Carcinog Risks Hum World Health Organ Int Agency Res Cancer. 2012;100(Pt E):1–538. PMC4781577 23193840

[8] WCRF/AICR. World Cancer Research Fund/ American Institute for Cancer Research. Diet, nutrition, physical activity and cancer: a global perspective. Continuous Update Project Expert Report 2018. WCRF/AICR. Available at: dietandcancerreport.org; 2018.

[9] Zhang YB, Pan XF, Chen J (2020). Combined lifestyle factors, incident cancer, and cancer mortality: a systematic review and meta-analysis of prospective cohort studies. Br J Cancer.

[10] Engel LS, Chow WH, Vaughan TL (2003). Population attributable risks of esophageal and gastric cancers. J Natl Cancer Inst.

[11] Wang SM, Katki HA, Graubard BI (2021). Population attributable risks of subtypes of esophageal and gastric cancers in the United States. Am J Gastroenterol.

[12] Steevens J, Schouten LJ, Goldbohm RA, van den Brandt PA (2010). Alcohol consumption, cigarette smoking and risk of subtypes of oesophageal and gastric cancer: a prospective cohort study. Gut.

[13] Wang Z, Koh WP, Jin A, Wang R, Yuan JM (2017). Composite protective lifestyle factors and risk of developing gastric adenocarcinoma: the Singapore Chinese Health Study. Br J Cancer.

[14] Wang X, Yang X, Li J (2019). Impact of healthy lifestyles on cancer risk in the Chinese population. Cancer.

[15] He Y, Bai Y, Wei S (2020). Healthy lifestyle and cancer risk among Chinese population in the Dongfeng-Tongji cohort. Ann Med.

[16] Jin G, Lv J, Yang M (2020). Genetic risk, incident gastric cancer, and healthy lifestyle: a meta-analysis of genome-wide association studies and prospective cohort study. Lancet Oncol.

[17] Kabat GC, Matthews CE, Kamensky V, Hollenbeck AR, Rohan TE (2015). Adherence to cancer prevention guidelines and cancer incidence, cancer mortality, and total mortality: a prospective cohort study. Am J Clin Nutr.

[18] Romaguera D, Vergnaud AC, Peeters PH (2012). Is concordance with World Cancer Research Fund/American Institute for Cancer Research guidelines for cancer prevention related to subsequent risk of cancer? Results from the EPIC study. Am J Clin Nutr.

[19] Buckland G, Travier N, Huerta JM (2015). Healthy lifestyle index and risk of gastric adenocarcinoma in the EPIC cohort study. Int J Cancer.

[20] Sheikh M, Poustchi H, Pourshams A (2019). Individual and combined effects of environmental risk factors for esophageal cancer based on results from the Golestan Cohort Study. Gastroenterology.

[21] Schulpen M, Peeters PH, van den Brandt PA (2019). Mediterranean diet adherence and risk of esophageal and gastric cancer subtypes in the Netherlands Cohort Study. Gastric Cancer.

[22] Li WQ, Park Y, Wu JW (2013). Index-based dietary patterns and risk of esophageal and gastric cancer in a large cohort study. Clin Gastroenterol Hepatol.

[23] van den Brandt PA, Goldbohm RA, van’t Veer P, Volovics A, Hermus RJ, Sturmans F (1990). A large-scale prospective cohort study on diet and cancer in The Netherlands. J Clin Epidemiol.

[24] Prentice RL (1986). A case-cohort design for epidemiologic studies and disease prevention trials. Biometrika.

[25] van den Brandt PA, Schouten LJ, Goldbohm RA, Dorant E, Hunen PM (1990). Development of a record linkage protocol for use in the Dutch Cancer Registry for Epidemiological Research. Int J Epidemiol.

[26] van den Brandt PA, van’t Veer P, Goldbohm RA (1993). A prospective cohort study on dietary fat and the risk of postmenopausal breast cancer. Cancer Res.

[27] Goldbohm RA, van den Brandt PA, Brants HA (1994). Validation of a dietary questionnaire used in a large-scale prospective cohort study on diet and cancer. Eur J Clin Nutr.

[28] Goldbohm RA, van’t Veer P, van den Brandt PA (1995). Reproducibility of a food frequency questionnaire and stability of dietary habits determined from five annually repeated measurements. Eur J Clin Nutr.

[29] Nevo-Table. Dutch Food Composition table 1986–1987; Nederlands voedingsstoffenbestand 1986–19871986

[30] Dirx MJ, Voorrips LE, Goldbohm RA, van den Brandt PA (2001). Baseline recreational physical activity, history of sports participation, and postmenopausal breast carcinoma risk in the Netherlands Cohort Study. Cancer.

[31] Fung TT, McCullough ML, Newby PK (2005). Diet-quality scores and plasma concentrations of markers of inflammation and endothelial dysfunction. Am J Clin Nutr.

[32] Mitrou PN, Kipnis V, Thiebaut AC (2007). Mediterranean dietary pattern and prediction of all-cause mortality in a US population: results from the NIH-AARP Diet and Health Study. Arch Intern Med.

[33] Trichopoulou A, Kouris-Blazos A, Wahlqvist ML (1995). Diet and overall survival in elderly people. BMJ.

[34] Trichopoulou A, Costacou T, Bamia C, Trichopoulos D (2003). Adherence to a Mediterranean diet and survival in a Greek population. N Engl J Med.

[35] Castro C, Peleteiro B, Lunet N (2018). Modifiable factors and esophageal cancer: a systematic review of published meta-analyses. J Gastroenterol.

[36] Fang X, Wei J, He X (2015). Landscape of dietary factors associated with risk of gastric cancer: a systematic review and dose-response meta-analysis of prospective cohort studies. Eur J Cancer.

[37] van den Brandt PA (2011). The impact of a Mediterranean diet and healthy lifestyle on premature mortality in men and women. Am J Clin Nutr.

[38] Gezondheidsraad. Health Council of the Netherlands. Physical Activity Guidelines 2017. The Hague: Gezondheidsraad; 2017.

[39] Gezondheidsraad. Health Council of the Netherlands. Dietary Guidelines 2015. The Hague: Gezondheidsraad; 2015.

[40] Lin D, Wei L (1989). The robust inference for the Cox proportional hazards model. J Am Stat Assoc.

[41] Schoenfeld D (1982). Partial residuals for the proportional hazards regression model. Biometrika.

[42] Xu JY, Vena JE, Whelan HK, Robson PJ (2019). Impact of adherence to cancer-specific prevention recommendations on subsequent risk of cancer in participants in Alberta's Tomorrow Project. Public Health Nutr.

[43] Brenner H, Gefeller O, Greenland S (1993). Risk and rate advancement periods as measures of exposure impact on the occurrence of chronic diseases. Epidemiology.

[44] Rockhill B, Newman B, Weinberg C (1998). Use and misuse of population attributable fractions. Am J Public Health.

[45] Newson RB (2013). Attributable and unattributable risks and fractions and other scenario comparisons. Stata J.

[46] Morze J, Danielewicz A, Przybylowicz K, Zeng H, Hoffmann G, Schwingshackl L (2021). An updated systematic review and meta-analysis on adherence to mediterranean diet and risk of cancer. Eur J Nutr.

[47] Ferro A, Morais S, Rota M (2018). Tobacco smoking and gastric cancer: meta-analyses of published data versus pooled analyses of individual participant data (StoP Project). Eur J Cancer Prev.

[48] Cook MB, Matthews CE, Gunja MZ, Abid Z, Freedman ND, Abnet CC (2013). Physical activity and sedentary behavior in relation to esophageal and gastric cancers in the NIH-AARP cohort. PLoS ONE.

[49] Moore SC, Lee IM, Weiderpass E (2016). Association of leisure-time physical activity with risk of 26 types of cancer in 1.44 million adults. JAMA Int Med..

[50] Chen P, Song Q, Han J (2017). Sitting time and occupational and recreational physical activity in relation to the risk of esophageal squamous cell carcinoma. Onco Targets Ther.

