# Supplementary material for: The impact of a healthy lifestyle on the risk of esophageal and gastric cancer subtypes
Source: Eur J Epidemiol. 2022 Aug 19;37(9):931–45. doi: 10.1007/s10654-022-00899-w (PMC9529711; doi:10.1007/s10654-022-00899-w)
Supplement: Supplementary file 1 — Supplementary file1 (PDF 401 kb) [file 10654_2022_899_MOESM1_ESM.pdf]

SUPPLEMENTARY MATERIALS to:

European Journal of Epidemiology

Article:

P.A. van den Brandt "The impact of a combined healthy lifestyle on the risk of esophageal and gastric cancer subtypes"

Supplementary Table S1. Definition of the combined healthy lifestyle score.

Supplementary Figure S1. Flow diagram of the number of subcohort members and cancer cases on which analyses are based, Netherlands Cohort Study (NLCS).

Supplementary Figure S2. Hazard ratios and 95% CIs (error bars) for the association between the healthy lifestyle score and risk of overall esophageal cancer in men and women separately, Netherlands Cohort Study (NLCS).

Supplementary Figure S3. Hazard ratios and 95% CIs (error bars) for the association between the healthy lifestyle score and risk of overall gastric cancer in men and women separately, Netherlands Cohort Study (NLCS).

Supplementary Figure S4. Spline regression curves for the association between healthy lifestyle score (HLS) and risk of A) overall esophageal cancer, and B) overall gastric cancer, Netherlands Cohort Study (NLCS).

Supplementary Figure S5. Hazard ratios and 95% CIs (error bars) for the association between risk of overall esophageal cancer and overall gastric cancer respectively, with each of the component lifestyle factors of the HLS (with mutual adjustment for the other component lifestyle factors), NLCS.

Supplementary Figure S6. Hazard ratios and 95% CIs (error bars) of esophageal and gastric cancer subtypes associated with a 1-point increment in healthy lifestyle score excluding smoking, in subgroups of tobacco smoking.

Supplementary Table S1. Definition of the combined healthy lifestyle score

Healthy lifestyle score (HLS): representing full, partial and noncompliance with the public health recommendation

Lifestyle factor	Compliance with public health recommendation		
	Full (1 point)	Partial (0.5 point)	No (0 point)
BMI (kg/m^2)	18.5- <25 (normal weight)	25- <30	≥ 30 or < 18.5
Smoking status	Never smoker	Former smoker	Current smoker
Physical Activity	Nonoccup PA > 60 min/day	> 30- ≤60 min/day	≤ 30 min/day
Diet: aMEDr	aMEDr: 6-8 points (high)	4-5 points (mod)	0-3 points (low)
Alcohol	≤ 10 g/day	> 10- <25 g/day	≥ 25 g/day

Abbreviations: aMEDr: alternate Mediterranean Diet Score excluding alcohol (range 0-8 points)

Minimum-Maximum score HLS: 0-5 (intervals 0.5)

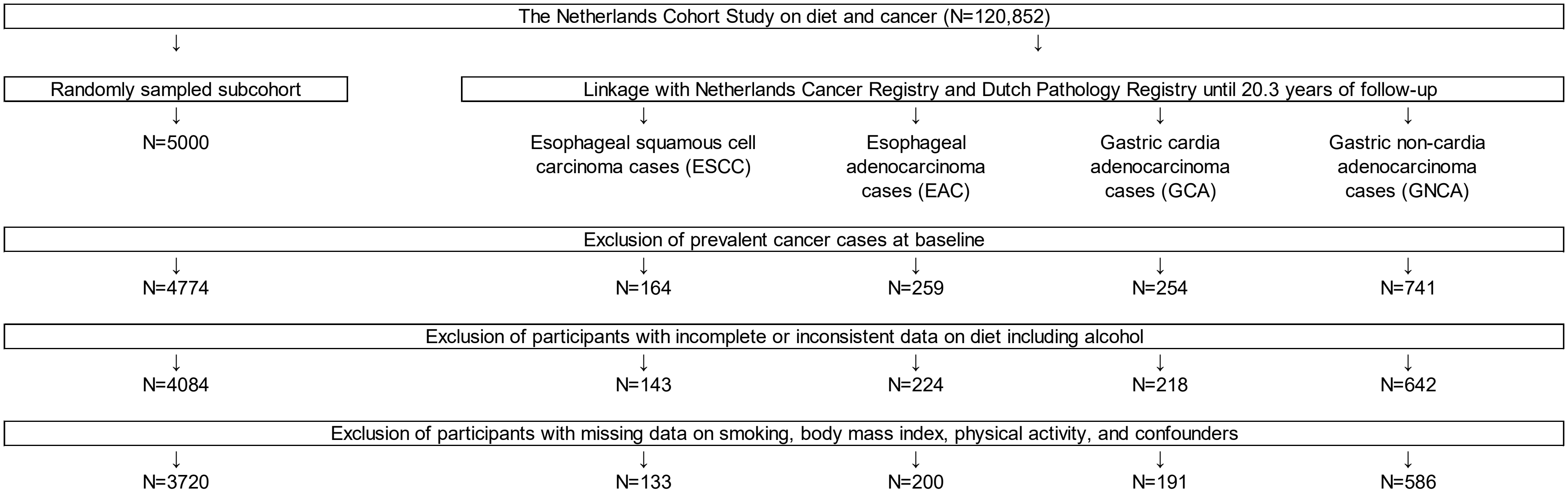

Supplementary Figure S1. Flow diagram of the number of subcohort members and cancer cases on which analyses are based, Netherlands Cohort Study (NLCS).

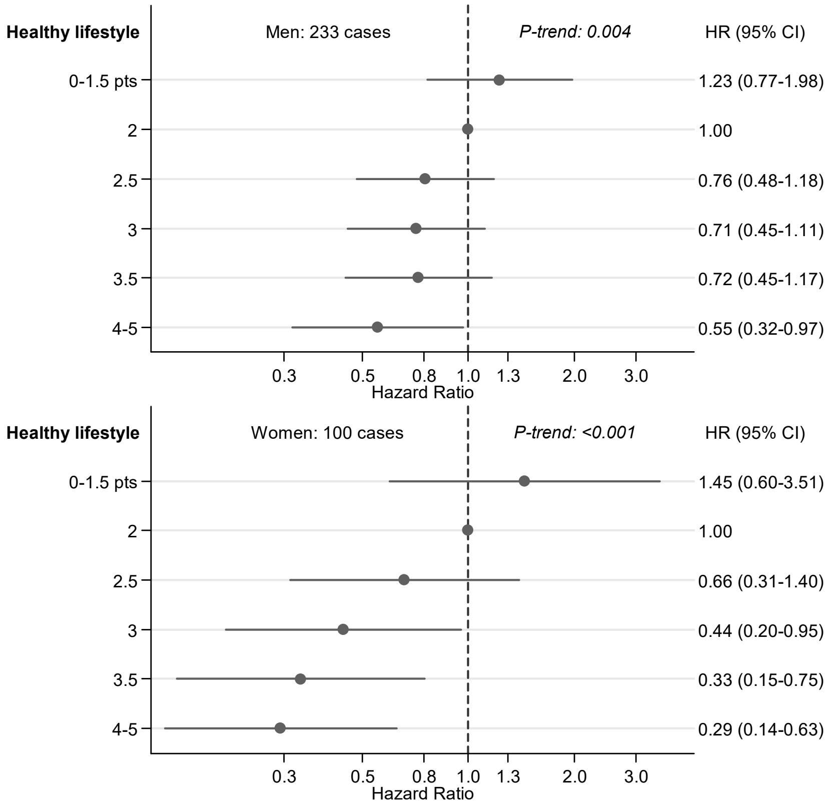

Supplementary Fig S2. Hazard ratios and 95% CIs (error bars) for the association between the healthy lifestyle score and risk of overall esophageal cancer in men and women separately, Netherlands Cohort Study (NLCS).

Multivariable analyses were adjusted for: age at baseline (years; continuous), cigarette smoking frequency (number of cigarettes per day; continuous, centered) and duration (number of years; continuous, centered)), highest level of education (primary school or lower vocational, secondary or medium vocational, and higher vocational or university), family history of esophageal cancer, chronic diseases at baseline: myocardial infarction, angina pectoris, stroke, hypertension, diabetes, asthma or bronchitis (no, yes), energy intake (continuous, kcal/day).

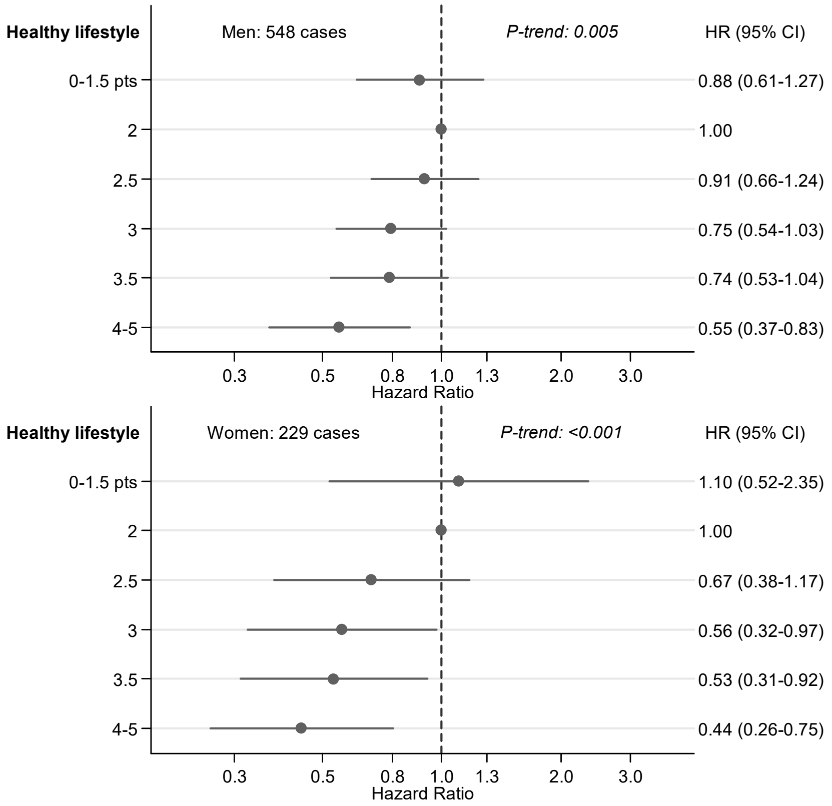

Supplementary Fig S3. Hazard ratios and 95% CIs (error bars) for the association between the healthy lifestyle score and risk of overall gastric cancer in men and women separately, Netherlands Cohort Study (NLCS).

Multivariable analyses were adjusted for: age at baseline (years; continuous), cigarette smoking frequency (number of cigarettes per day; continuous, centered) and duration (number of years; continuous, centered)), highest level of education (primary school or lower vocational, secondary or medium vocational, and higher vocational or university), family history of gastric cancer, chronic diseases at baseline: myocardial infarction, angina pectoris, stroke, hypertension, diabetes, asthma or bronchitis (no, yes), energy intake (continuous, kcal/day).

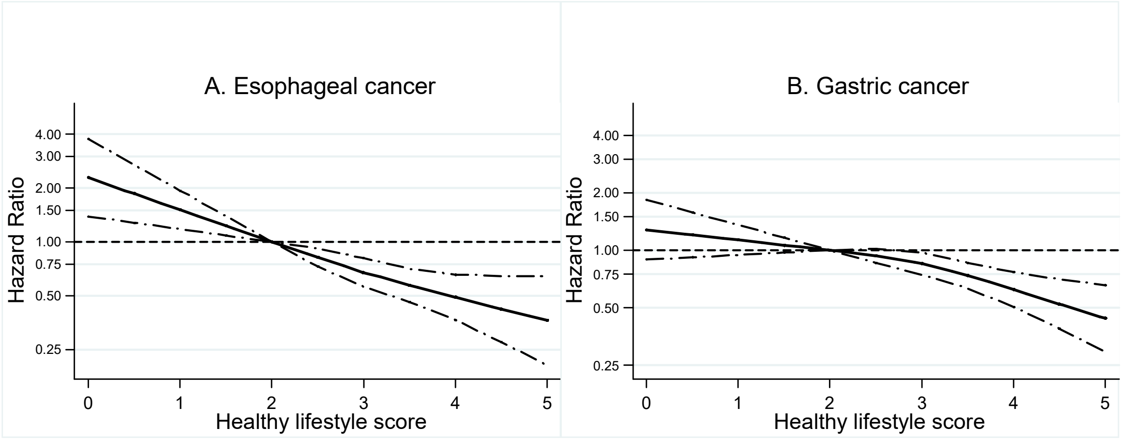

Supplementary Fig S4. Spline regression curves for the association between healthy lifestyle score (HLS) and risk of A) overall esophageal cancer, and B) overall gastric cancer, Netherlands Cohort Study (NLCS). Solid lines represents point estimates and dashed lines represent 95% confidence intervals.
Multivariable HRs were calculated by restricted cubic spline regression (using 3 knots) adjusting for: age at baseline (years; continuous), sex, cigarette smoking frequency (number of cigarettes per day; continuous, centered) and duration (number of years; continuous, centered)), highest level of education (primary school or lower vocational, secondary or medium vocational, and higher vocational or university), family history of esophageal cancer, family history of gastric cancer (respectively), chronic diseases at baseline: myocardial infarction, angina pectoris, stroke, hypertension, diabetes, asthma or bronchitis (no, yes), energy intake (continuous, kcal/day). P-values for non-linearity tests were 0.667 for esophageal cancer and 0.219 for gastric cancer.

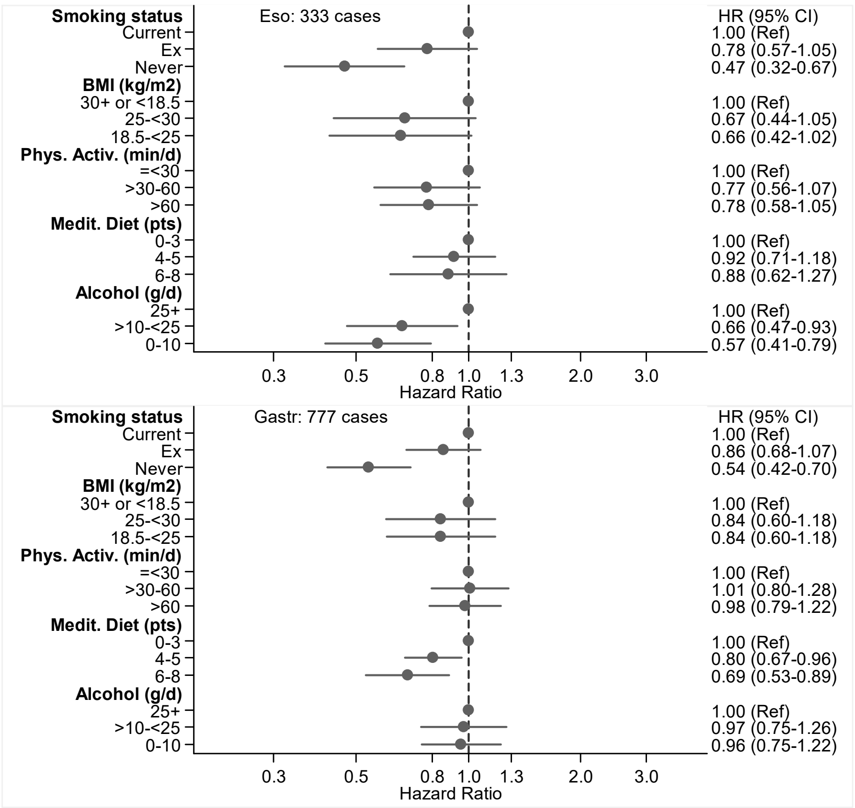

Supplementary Fig S5. Hazard ratios and 95% CIs (error bars) for the association between risk of overall esophageal cancer and overall gastric cancer respectively, with each of the component lifestyle factors of the HLS (with mutual adjustment for the other component lifestyle factors), Netherlands Cohort Study (NLCS).

Multivariable HRs were adjusted for: age at baseline (years; continuous), sex, cigarette smoking frequency (number of cigarettes per day; continuous, centered) and duration (number of years; continuous, centered)), highest level of education (primary school or lower vocational, secondary or medium vocational, and higher vocational or university), family history of esophageal cancer, family history of gastric cancer (respectively), chronic diseases at baseline: myocardial infarction, angina pectoris, stroke, hypertension, diabetes, asthma or bronchitis (no, yes), energy intake (continuous, kcal/day), other component lifestyle factors of the HLS.

P-values for trend tests were for esophageal cancer: <0.001 for smoking status, 0.191 for BMI, 0.170 for physical activity, 0.431 for Mediterranean Diet adherence, 0.001 for alcohol.

For gastric cancer, P-values for trend tests were: <0.001 for smoking status, 0.551 for BMI, 0.813 for physical activity, 0.001 for Mediterranean Diet adherence, 0.719 for alcohol.

Abbreviations: Eso, esophageal cancer; Gastr, gastric cancer.

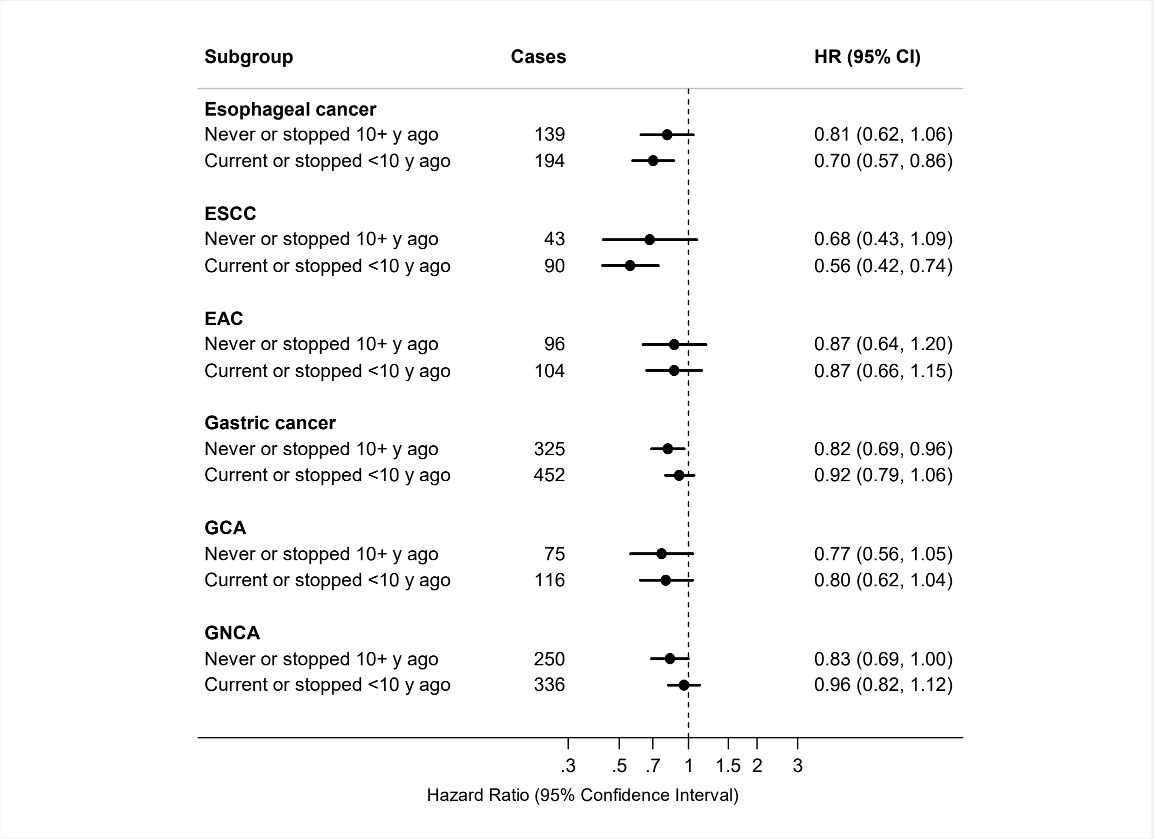

Supplementary Fig S6. Hazard ratios and 95% CIs (error bars) of esophageal and gastric cancer subtypes associated with a 1-point increment in healthy lifestyle score excluding smoking, in subgroups of tobacco smoking.
Multivariable analyses were adjusted for: age at baseline (years; continuous), sex, cigarette smoking frequency (number of cigarettes per day; continuous, centered) and duration (number of years; continuous, centered)), highest level of education (primary school or lower vocational, secondary or medium vocational, and higher vocational or university), family history of gastric cancer, chronic diseases at baseline: myocardial infarction, angina pectoris, stroke, hypertension, diabetes, asthma or bronchitis (no, yes), energy intake (continuous, kcal/day).
Abbreviations: ESCC, esophageal squamous cell carcinoma; EAC, esophageal adenocarcinoma; GCA, gastric cardia adenocarcinoma; GNCA, gastric non-cardia adenocarcinoma.
